# Microwave-assisted synthesis, characterization and in vitro biomedical applications of *Hibiscus rosa-sinensis* Linn.-mediated carbon quantum dots

**DOI:** 10.1038/s41598-024-60726-y

**Published:** 2024-04-30

**Authors:** Shweta Yalshetti, Bothe Thokchom, Santosh Mallikarjun Bhavi, Sapam Riches Singh, Sneha R. Patil, B. P. Harini, Mika Sillanpää, J. G. Manjunatha, B. S. Srinath, Ramesh Babu Yarajarla

**Affiliations:** 1 Drosophila and Nanoscience Research Laboratory, Department of Applied Genetics, Karnatak University, Dharwad, Karnataka 580003 India; 2 Department of Zoology, Bangalore University, Bangalore, Karnataka 560056 India; 3 Department of Biological and Chemical Engineering, Aarhus University, Norrebrogade 44, 8000 Aarhus C, Denmark; 4 Department of Chemistry, FMKMC College, Mangalore University Constituent College, Madikeri, Karnataka 571201 India; 5 Department of Microbiology and Biotechnology, Bangalore University, Bangalore, Karnataka 560056 India

**Keywords:** *Hibiscus rosa-sinensis*, Carbon quantum dots (CQDs), Antibacterial, Anti-inflammatory, Wound healing, Nanoscale materials, Biological techniques, Nanoscience and technology

## Abstract

In recent years, carbon quantum dots (CQDs) have garnered considerable attention as a promising material for biomedical applications because of their unique optical and biological properties. In this study, CQDs were derived from the leaves of *Hibiscus rosa-sinensis* Linn. via microwave-assisted technique and characterized using different techniques such as ultraviolet–visible, Fourier transform infrared, fluorescence spectrometry, X-ray diffraction, dynamic light scattering, transmission electron microscopy and energy-dispersive X-ray spectroscopy. Subsequently, their potential for biomedical applications was investigated through in vitro assays assessing scratch healing, anti-inflammatory, antibacterial, and cytotoxicity properties. It was found that the CQDs were fluorescent, polycrystalline, quasi-spherical, ~ 12 nm in size with presence of –OH and –COOH groups on their negatively charged surfaces, and demonstrated good anti-inflammatory by inhibiting protein denaturation, cyclooxygenase-2 and regulating inflammatory cytokines. The CQDs also exhibited antimicrobial activity against *Klebsiella pneumoniae* and *Bacillus cereus,* good biocompatibility, along with excellent promotion of cell proliferation in vitro, indicating their potential as a anti-inflammatory and wound healing material. The properties were more enhanced than their precursor, *H. rosa-sinensis* leaf extract. Hence, the CQDs synthesized from the leaves of *H. rosa-sinensis* can serve as a potential biomedical agent.

## Introduction

In the recent years, carbonaceous or carbon-based nanoparticles have gained immense attention because of their potential properties in biocompatibility, photoluminescence, electrochemical properties^1^, and their practical applicabilities like bioimaging^2^, sensing^3^, DNA and drug delivery^4,5^, etc. Since their accidental discovery in 2004^6^, carbon quantum dots (CQDs) have been extensively studied in the fields of optoelectronics and biomedicine. CQDs have been developed through two primary approaches, “top-down” and “bottom-up”, based upon the breaking or making order of precursor molecules to obtain the quantum dots. The top-down approaches such as electrochemical carbonization^7^, laser ablation^8^ and chemical ablation^9^ methods are multi-step processes with poor quantitative yield. Whereas the bottom-up approaches like hydrothermal, microwave-assisted and thermal decomposition methods are one-step processes that tend to be more advantageous^10^. Hence, CQDs synthesized via bottom-up approach vary with the one synthesized from top-down approach in optical properties and their applications as well. This variation depends on the synthetic method and the source of carbon precursor used^10^. The size, shape, functional groups and surface doping of CQDs play a crucial role in determining their optical properties such as light absorption, light emission as well as their efficiency in multiphoton up-conversion and down-conversion^11^. Generally, the CQDs’ surface contain oxygenated functional groups like hydroxyl, carboxylic acid, carbonyls and epoxy groups^12^, which is responsible for biocompatibility and good solubility of CQDs by imparting them polarity.

The traditional synthesis of CQDs involves the use of toxic and hazardous chemicals, such as organic solvents, strong acids, and bases, which can pose a risk to human health and the environment. In contrast, green synthesis methods use natural, renewable, and biodegradable resources, such as waste biomass^13^, plant extracts^14^, fruit juices^15^, and microorganisms^16^, to produce CQDs without the need for toxic chemicals, which ensures a more promising sustainable and eco-friendly alternative. Thus, fabricating and functionalizing CQDs with medicinal plants is a new progress which has potential in the field of biomedical diagnosis. Medicinal plants like neem^17^, turmeric^18^, ginger^19^, aloe vera^20^, tulsi^21^, brahmi^14^ and many others belong to this list. This combinational study cumulates the unique properties of plant and CQDs and enhance their applicability. So formed CQDs may act as an excellent substitute to many chemical fluorescent probes which are toxic, less biocompatible, less photostable and photo bleachable. The functionalities of the plant impart medicinal properties such as healing, anti-inflammatory, antioxidant, etc. One such medicinal plant is the *Hibiscus rosa-sinensis* Linn., extensively used in the folk medicinal practices.* H. rosa-sinensis,* a shrub of Malvaceae family, is native to China^22^. Many species have been extensively cultivated in tropical regions as an ornamental plant with varying colours of flowers under relatively high humid conditions. *H. rosa-sinensis* made its place in folk medicinal systems because of its range of properties like anti-greying, antifertility^23,24^, hair growth promotion^25^, treatment of ulcer, stomach pain, anti-cancer^26,27^, anti-inflammatory and central nervous system depressant^28^. These properties are exhibited by most parts of the plant, the phytochemistry of the leaves display the presence of tannins, flavonoids, steroids, alkaloids, saponins and anthocyanins majorly, acting as organic carbon precursor compounds^29^. Hence, the leaves of this plant can be considered to be used as the carbon precursor of this study.

Wound healing, a critical and fundamental process, is vital for restoring tissue function and maintaining homeostasis. However, various diseases and disorders can obstruct or delay this process, resulting in chronic wounds that heal slowly and pose significant risks of morbidity and mortality. Chronic wounds can arise from a variety of underlying conditions, including diabetes, peripheral vascular disease, and autoimmune disorders^30^. These conditions can impair the normal inflammatory response, angiogenesis, and extracellular matrix deposition, leading to a prolonged and ineffective healing process^31^. Additionally, bacterial infections frequently complicate chronic wounds, exacerbating the underlying tissue damage and impairing the healing process. Despite significant advances in medical science, the treatment of chronic wounds remains a significant clinical challenge, highlighting the need for novel therapeutic strategies. In this context, carbon quantum dots synthesized using *H. rosa-sinensis* leaves have become a promising approach for enhancing wound healing.

The present work focuses on preparation of CQDs via cost effective, single-step microwave-assisted method from the leaf extract of *H. rosa-sinensis.* The size, shape, stability, elemental composition, optical properties and surface functionalities were measured using different characterization techniques. It also concentrates on the biological applications via various in vitro analyses, unravelling the efficiency of the CQDs in wound healing alongside its anti-inflammatory and antimicrobial properties.

## Experimental

### Chemicals and materials

Healthy leaves of cultivated *Hibiscus rosa-sinensis* Linn. were obtained from a home garden in Dharwad, Karnataka, India (15°27′36.9072″N, 75°0′37.0224″E). It was then identified and authenticated by Dr. Shivanand S. Bhat, Taxonomist, Smt. Indira Gandhi Government First Grade Women’s College, Sagar, Karnataka, India (Specimen Acc. No. IGGFWC/Hib-042). The collection of the plant material and the research work associated with this complies with relevant institutional, national, and international guidelines and legislation. Gram positive- *Bacillus cereus* and Gram negative- *Klebsiella pneumoniae* bacteria were acquired from the Department of Microbiology, Karnatak University Dharwad, Karnataka, India. Mouse fibroblastic L929 (NCTC Clone 929) and human keratinocytic HaCaT cells were procured from National Centre for Cell Science, Pune, Maharastra, India. GENLISA™ Human IFN-$\upgamma$, IL-6, TNF-α β, IL-10 and IL-1β were procured from Krishgen Biosystems. DMEM (Dulbecco’s Modified Eagle Medium) was purchased from Sigma Aldrich Co. LLC. Bovine serum albumin (BSA), dimethyl sulfoxide (DMSO), 3-(4,5-dimethylthiazol-2-yl)-2,5-diphenyltetrazolium bromide (MTT reagent), N,N,N′,N′-Tetramethyl-p-phenylenediamine (TMPD) and nutrient broth were obtained from HiMedia Lab. Bovine hemin chloride and arachidonic acid were bought from SRL chemicals. The chemicals and reagents involved in this study were analytical grade and employed as such without additional processing or treatment.

### Preparation of plant extract

The collected leaves were washed frequently with distilled water and air-dried. The dry leaves were weighed and chopped to fine pieces. Plant extract was prepared by adding 10 g of leaf powder to 100 mL of distilled water and autoclaving at 121 °C, 30 psi for 20 min. Autoclaving ensures the elimination of contaminants such as microorganisms and fungal spores. Subsequently, the mixture was filtered through muslin cloth and then through Whatman filter paper (grade 1).

### Green synthesis of CQDs using microwave assisted method

The synthesis of CQDs was carried out as per a previously reported simple microwave-assisted method, with slight modification^14^. 20 mL of aqueous leaf extract was taken in a 250 mL conical flask and irradiated in the microwave oven (IFB 20PG3S) for 30 s, while avoiding spillage, and cooled for 1 min. This process was repeated until the light green extract converts to dark brown CQDs (~ 20 min). The crude CQDs was subjected to 30 min centrifugation at 5000 rpm. The supernatant was retrieved and filtered through a 0.22 µm microfilter and stored at 4 °C. The CQDs solution was lyophilized to powder for future use.

### Physico-chemical characterizations

The JASCO V-670 UV–VIS-NIR spectrometer with wavelength range of 200–800 nm, was used to obtain the ultraviolet–visible (UV–Vis) absorption spectrum of CQDs. Meanwhile, Hitachi F-700 Fluorescence spectrometer with excitation wavelength ranging from 300 to 500 nm was utilized to measure the fluorescence emission spectra. Rigaku Smartlab SE was employed to acquire the X-ray diffraction (XRD) pattern through irradiation of Cu Kα ($\uplambda$ = 0.15406 nm) at 30 mA and 40 kV, and 40° min^−1^ scan rate. Energy-dispersive X-ray (EDX) spectroscopy was performed with JOEL JSM-IT500 to check the purity and elemental composition of the CQDs. Horiba SZ-100 was harnessed for measuring the zeta potential and the hydrodynamic size of the particles in solution by dynamic light scattering (DLS). Confirmation of particle size and morphological study was done using transmission lectron microscopy (TEM - JEOL JEM-2100 Plus). Fourier transform-infrared spectroscopy (FT-IR) was performed over the range of 4000–﻿400 cm^-1^ using Nicolet iS10 FTIR spectrophotometer.

### In vitro biomedical applications

#### Cytotoxicity test

Cytotoxicity of the synthesized CQDs and plant extract was analyzed by MTT assay using L929 and HaCaT cell lines. Cells were cultured in DMEM medium, with approximately 10,000 seeded cells per well in a 200 μL suspension, followed by incubation at 37 °C and 5% CO_2_ atmosphere for 24 h. Thereafter, the cells were treated with different concentrations (100, 200, 300, 400 and 500 μg mL^−1^ for L929 cells; 1, 10, 50, 100, 250, 500, 1000 µg mL^−1^ for HaCaT cells) of CQDs and plant extract along with standard drug (Cisplatin = 15 μg mL^−1^)) which was followed by incubation at 37 °C for 24 h in 5% CO_2_, treatment of 10% MTT reagent and final incubation for 3 h. Further, 100 μL of solubilization solution (DMSO) was introduced to the formazan that had formed, and the absorbance was assessed by means of a microplate reader at 570 nm, with 630 nm serving as the reference wavelength, to quantify the viable cells. The percentage growth inhibition and median lethal concentration (LC_50_) were derived from the dose–response curves.

#### Apoptosis assay

The effects of CQDs in the cellular apoptosis of HaCaT cells were assessed using Annexin V and propidium iodide (PI) staining, followed by flow cytometry^32^. Cells were treated with CQDs (250 µg mL^−1^) and cultured for 24 h, while cells without treatment were considered as control. Following this, the cells were washed twice with cold phosphate buffer saline (PBS) and resuspended in 1X﻿ binding buffer at a concentration of 1 × 10^6^ cells mL^−1^. Subsequently, the cells were divided into groups including unstained cells, control group, Annexin only group, PI only group, and CQDs group. Annexin V-FITC and PI were added to their respective labelled tubes. After vortexing and incubation for 15 min at room temperature, 1X binding buffer was supplemented to each tube. The samples were then analyzed using a Flow Cytometer (BD FACS LyricTM) within 1 h.

#### Hemolysis assay

The hemolytic properties of CQDs were evaluated using in vitro hemolysis assay as reported, with slight modification^33^. Goat blood was centrifuged (1000×*g*, 15 min, 4 °C) to remove plasma and buffy coat, and erythrocytes were washed thrice and suspended in PBS. 500 µL of 1% erythrocyte suspension was incubated with 100 µL of different concentrations of CQDs (1–1000 µg mL^−1^) for 30 min at 37 °C, after which 400 µL of PBS was added and absorbance was measured after centrifugation. PBS and 10% RBC lysis buffer were used as negative and positive controls respectively.

#### Wound healing property

The wound healing property of the CQDs was studied via standard in vitro scratch wound healing assay, with slight modifications, using L929 and HaCaT cell lines ^34^. The cells were cultured at 37 °C and 5% CO_2_ atmosphere for 24 h to reach ~ 100% confluence as a monolayer and a scratch was made perpendicular to the bottom of the well of microplate. The scratch or the cell gap was washed twice with DMEM media and with pH 7.4 PBS. Subsequently, the cells were treated with a mixture of 1 mL fresh medium and 25 μL specified concentrations of CQDs (31.40 μg mL^–1^ for L929; 250 μg mL^−1^ for HaCaT), plant extract (34.60 μg mL^−1^) and standard ascorbic acid (15 μg mL^−1^) respectively. The wound closure was recorded at different time points of 0 h, 12 h and 24 h with subsequent incubation. The photos of the wound at different time intervals were analyzed using ‘Wound_healing_size_tool’ plugin in imageJ with a variance window size of 20, threshold value of 100 and percentage of saturated pixels of 0.0001, to assess the progress of healing. The percentage of wound closure was calculated using the formula:

$$Wound \;Closure \%=\left(\frac{{A}_{t=0}-{A}_{t=\Delta t}}{{A}_{t=0}}\right)\times 100$$

where ${A}_{t=0}$ and ${A}_{t=\Delta t}$ are the wound areas at 0 and n hours after initial scratch.

### Protein denaturation assay

The anti-inflammatory properties of the CQDs were initially analyzed via protein denaturation assay^35^, with some changes. A sample mixture containing 1 mL of PBS, 50 µL of BSA and different saturations (50, 100, 150, 200, 250 µg mL^−1^) of CQDs, plant extract and standard (aspirin) was concocted, and left to incubate at room temperature for a duration of 15 min. It was then incubated for 15 min in a hot water bath at 70 °C, resulting in the denaturation of proteins. The absorbance at 660 nm was appraised using Labman UV Visible Spectrophotometer, and the degree of protein denaturation inhibition or anti-inflammatory action was determined by applying the formula:

$$\% Protein\;denaturation\;inhibition = \frac{{\left( {{\text{Absorbance}}\;{\text{of}}\;{\text{control}} - {\text{Absorbance}}\;{\text{of}}\;{\text{test}}\;{\text{sample}}} \right)}}{{({\text{Absorbance}}\;{\text{of}}\;{\text{control}})}} \times 100$$

### ELISA based inflammatory cytokine expression assay

The expression of pro- and anti-inflammatory cytokines (IFN-$\upgamma$, TNF-α, IL-6, IL-10 and IL-1β) were assessed using ELISA as per kit method on HaCaT cells.

### Cyclooxygenase-2 (COX-2) inhibition assay

To 970 µL of reaction buffer containing Tris:heme:phenol (100 mM:1 µM:1 µM), 20 µL of different concentrations of CQDs (1, 10, 50, 100, 125, 250 and 500 µg mL^−1^) and positive control, celecoxib (0.78, 1.56, 3.125, 6.25, 12.5, 25 and 50 µg mL^−1^) in Tris HCl buffer (100 mM, pH 8.0) were added. The reaction was initiated by adding 5 µL arachidonic acid (10 mM) and 5 µL TMPD solution (17 mM). The reaction was allowed to take place at room temperature for 10 min, after which absorbance was observed at 595 nm (iMark microplate reader, BioRad). The median inhibitory concentration (IC_50_) was calculated using the dose-dependent curve.

### Antimicrobial analysis

Two strains of bacterial cultures were chosen to determine the antimicrobial activity of synthesized CQDs. Gram positive- *Bacillus cereus* and Gram negative- *Klebsiella pneumoniae* bacteria were collected and cultured initially on nutrient agar medium, incubated overnight and subsequently stored.

Agar well diffusion method was opted for the antimicrobial analysis of CQDs and plant extract using kanamycin (50 µg) as standard control. Bacterial cultures with optical density value of 1 at 600 nm (OD_600_) were spread across nutrient agar plates uniformly. Wells were bored using well borer and different doses of CQDs and plant extract (100 µg, 125 µg, 150 µg), in DMSO, were added to the wells. Following that, the plates were subjected to an overnight incubation at a temperature of 37 °C. The antibacterial activity was subsequently assessed by measuring the diameter of the inhibition zone surrounding the well.

The antibacterial properties of the samples were further evaluated by colony counting assay. Bacteria were inoculated into nutrient agar along with different concentrations of CQDs and plant extract (100 µg mL^−1^, 125 µg mL^−1^, 150 µg mL^−1^), and standard kanamycin (50 µg mL^−1^), and cultured for 12 h at 37 °C, after which they were diluted 500 times and spread on agar plates. The plates were incubated for another 12 h at 37 °C and the active colonies were compared.

### Statistical analysis

Utilizing GraphPad Prism 8 software, statistical analysis was performed by one-way ANOVA followed by post hoc Tukey HSD test, for statistical relevance among groups (*p* < 0.05). The findings were reported as the mean $\pm$ standard error of the mean (SEM) or mean ± standard deviation (SD), wherever applicable.

## Results and discussion

### Synthesis of CQDs

Phytochemicals and phytohormones are the predominant component of leaf extract biomass along with polymers like cellulose, hemicellulose, and lignin, such biopolymers are abundant source of carbon for the synthesis of CQDs. Moreover, they are rich in hydroxyl, carbonyl, and epoxy groups. Hydrolysis of such large compounds lead to the formation of smaller counterpart, which act as precursors in bottom-up method. Further, microwave irradiation under high pressure and temperature above their melting points lead to the series of complex dehydrogenation and condensation reactions converting biomass readily into CQDs^36^.

**Figure 1:**
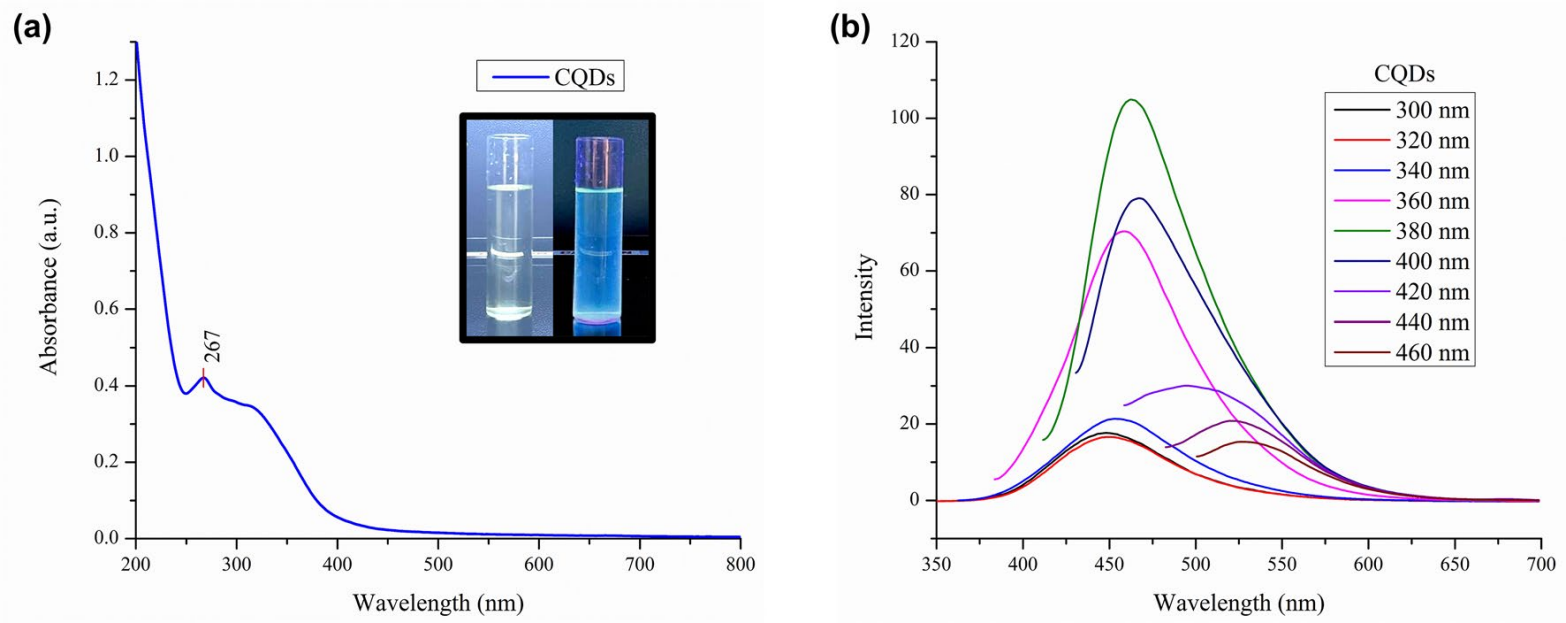
Optical properties of synthesized CQDs: (a) UV–Visible absorption spectrum (inset: photographs of CQDs in response to white light and UV); (b) Fluorescence emission spectra of CQDs across $\uplambda$_ex_ = 300–460 nm at 20 nm intervals.

*H. rosa-sinensis* contain phytochemicals such as flavonoids, tannins, saponins, steroids, phenols, alkaloids, pro-anthocyanidin, cyanidin-3-sophoroside-5-glucoside, cyanidin-3,5-diglucoside, quercetin-3,7-diglucosidem^29^. The leaves were found to contain a variety of fatty acids, alcohols and hydrocarbons, such as tridecanoic acid, pentadecanoic acid, undecanoic acid, tricosanoic acid, triacontan-1-ol, tricosan-1-ol, stearic acid, tartaric acid and pentacosanoic acid^28^. These molecules undergo various reactions of carbonization via hydrolysis, dehydration, and decomposition followed by polymerization, condensation and cycloaddition reactions^37^, eventually generating the carbon core, which gets passivated and functionalised leading to the formation of the CQDs. CQDs synthesis was primarily indicated by the colour change of extract solution from light green to dark brown and confirmed by the visible blue fluorescence under UV light (Fig. 1b (inset)).

### Optical properties of CQDs

UV–Vis spectrometry unveiled the presence of an absorption peak at 267 nm (Fig. 1a), accompanied by a tail that extended into the visible region. This absorption pattern corresponds to electron shift in the C=C bonds (π − π* interaction) and the C=O bond (n–π^*^ interaction) of the aromatic sp^2^ domains in the carbon core^38^. In comparison, similar peaks at 270 and 320 nm were obtained by Tripathi et al.^39^, confirming the occurrence of electronic shifting attributed to the presence of C=C and C=O functional groups on the CQD’s surface.

The fluorescence studies of CQDs exhibited fluorescence for excitation wavelengths of 300 to 460 nm range. The highest emission was observed at 460 nm (blue colour) corresponding to the excitation at 380 nm (Fig. 1b) due to a large number of particles being excited at that wavelength. However, a bathochromic shift (red shift) was observed as the excitation wavelength increased from 420 nm. This shift can be attributed to the presence of various functional groups which act as auxochromes and chromophores. This excitation dependent emission depends upon various aspects such as particle size, surface energy traps and functional groups^40^.

### Elemental, structural and surface analyses of CQDs

**Figure 2:**
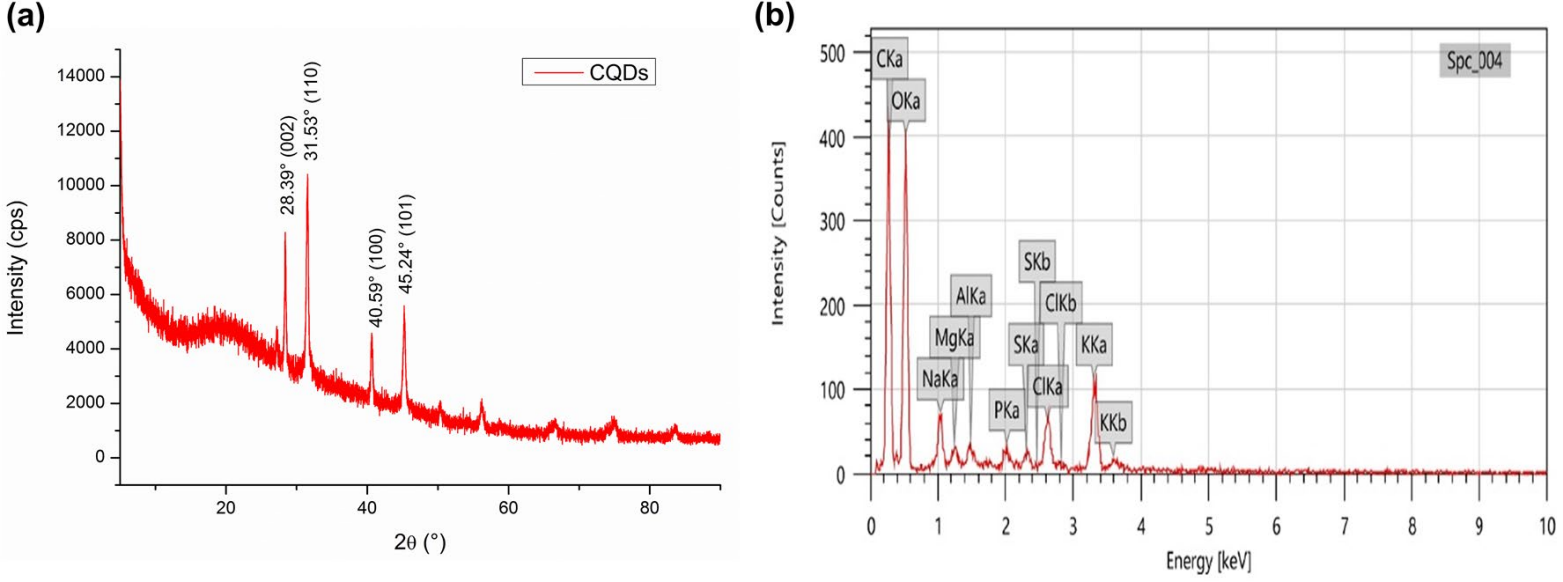
Elemental properties of synthesized CQDs: (a) XRD pattern; (b) EDX spectrum.

The XRD analysis of CQDs (Fig. 2a) revealed distinct sharp peaks at 2θ = 28.39°, 31.53°, 40.59° and 45.24°. Bragg’s equation was employed to calculate the d-spacing values (d) for the CQDs:

$${\text{n }}\lambda = {\text{ 2d sin }}\theta$$

where n is a positive integer usually considered to be 1 for first order diffraction peaks, λ denotes the incident X-rays’ wavelength, specifically 0.15406 nm, and θ represents the Bragg’s angle of diffraction. The d-spacing values of CQDs at 2θ = 28.39°, 31.53°, 40.59° and 45.24° were calculated to be 3.14 Å, 2.83 Å, 2.22 Å and 2.00 Å respectively which is proportional to the (0 0 2), (1 1 0), (1 0 0) and (1 0 1) planes of graphitic carbon respectively^41,42^, thus, revealing a polycrystalline nature. Further, the weak peaks at 2θ = 56.18°, 66.58° and 83.57° may be distorted peaks corresponding to the (0 0 4), (1 0 3) and (1 1 2) planes of crystalline carbon lattice (JCPDS card no. 01–086-8298). These distortions can be due to the interaction of carbon with oxygen, which acts as a dopant because of its abundance in the plant extract^43^.

The elemental composition and purity of CQDs were disclosed by EDX analysis (Fig. 2b), wherein, carbon and oxygen were the major elements with 48.9 wt% and 45.93 wt% respectively. However, small traces of impurities of Na, Mg, Al, S, Cl were also present with total abundance of 5 wt% which is similar with other reported CQDs^44^.

**Figure 3:**
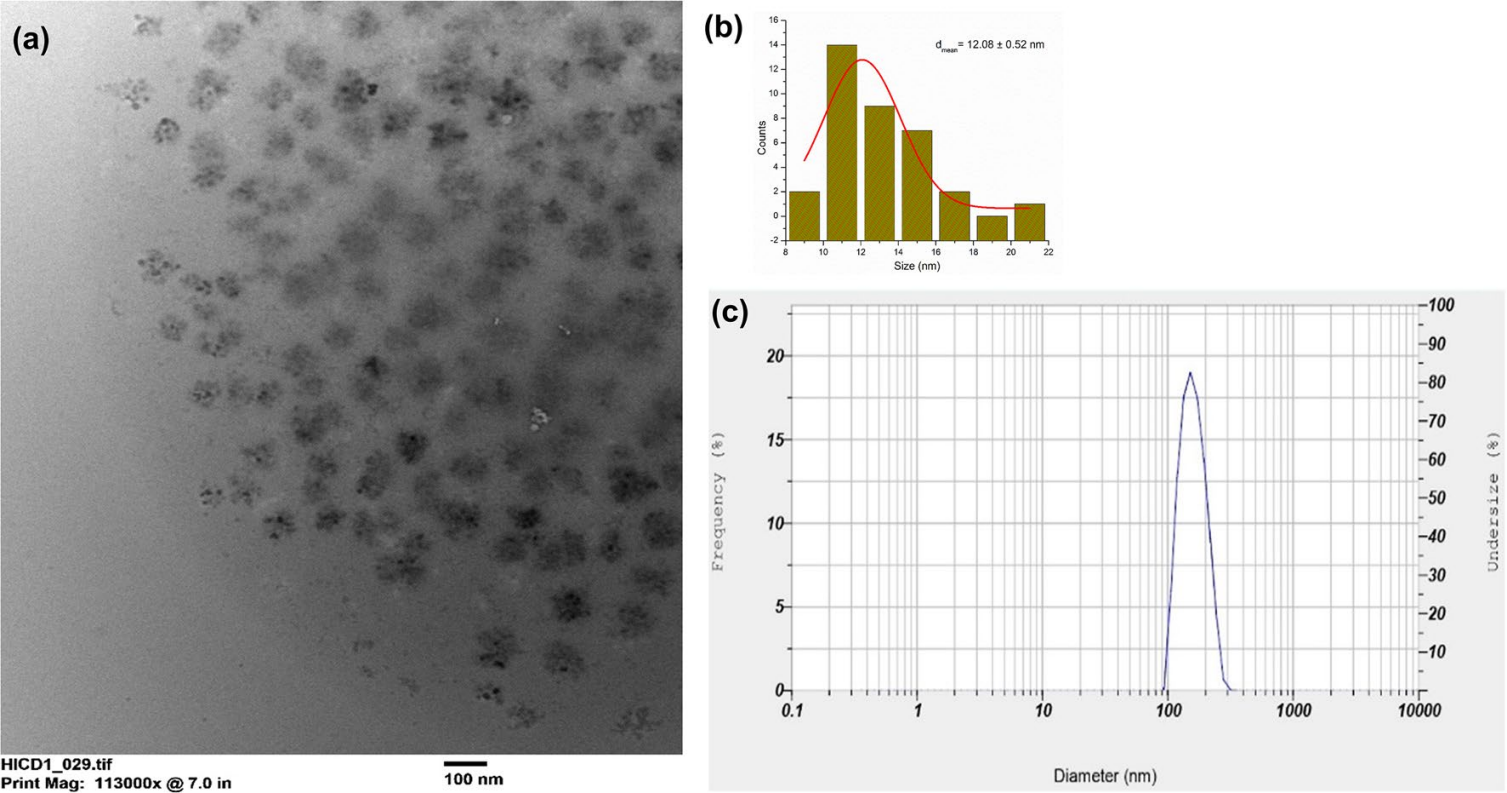
The structural and size distribution of CQDs: (**a**) TEM image; (**b**) grain size histogram of TEM; (**c**) particle size via DLS.

The sizes of the CQD particles were assessed using DLS and TEM, and the average diameters were found to be 113.5 nm and 12.08 $\pm$ 0.52 nm respectively. The TEM statistics was obtained in the dry state of CQDs sample (Fig. 3a,b) which is in its most compact and least interacted state, while DLS measurements for CQDs were acquired in its solvated form (Fig. 3c) which involves the association of solvent molecules (water) with the functional groups of the CQDs via different non-covalent interactions like hydrogen bonding and van der Waals interactions. This might have led to the formation of larger diffusion layer resulting in larger hydrodynamic size than TEM^45^. Another reason could be the weak electrostatic repulsion and less steric stabilization which can be justified by the zeta potential analysis.

**Figure 4:**
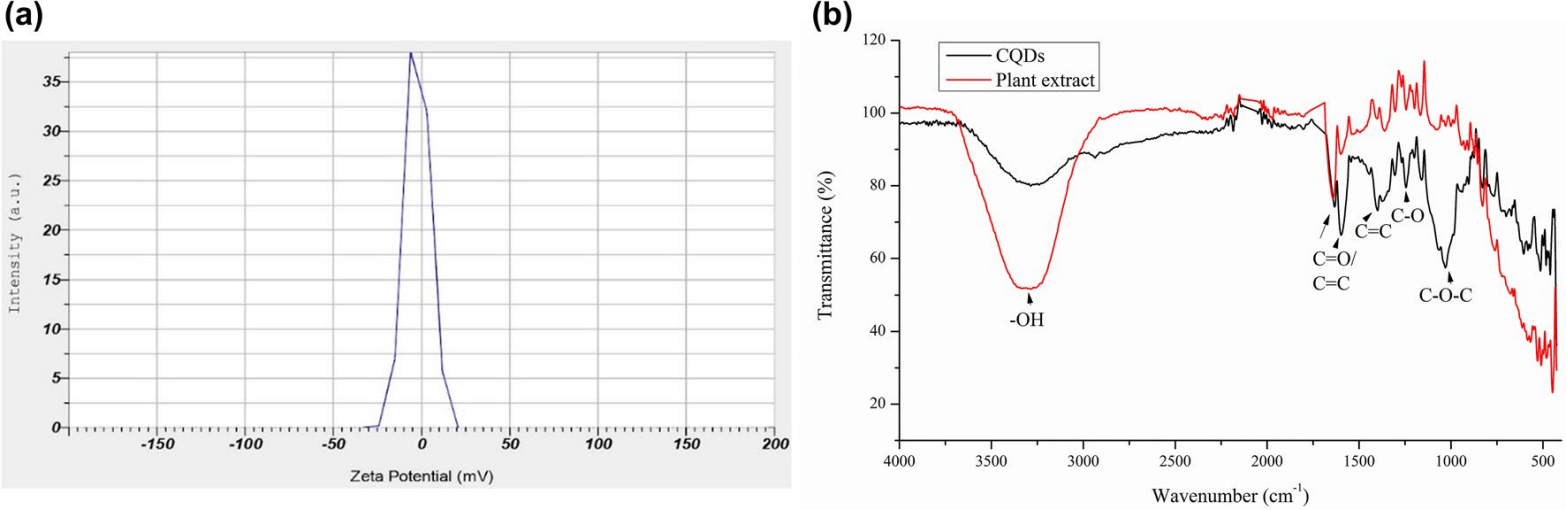
Surface analysis of CQDs: (**a**) zeta potential; (**b**) FT-IR spectra (alongside plant extract).

Zeta potential analysis revealed that the CQDs have a net negative surface charge of -2.1 mV (Fig. 4a). Zeta potential corresponds to the stability and surface charge of the colloidal CQDs, in which greater positive or negative surface charge exhibiting molecules tend to repel each other and are considered more stable, whereas, lesser tend to agglomerate due to less repulsive force exerted by them.

The presence of functional groups on the surface of the synthesized CQDs were determined by the FT-IR analysis (Fig. 4b). The obtained FT-IR spectra exhibited peaks corresponding to the stretching or bending of different chemical bonds. The broad peak at 3285 cm^−1^ can be attributed to O–H stretching vibrations. The strong peaks at 1632 cm^−1^ and 1597 cm^−1^ can be assigned to C=O^46^ or C=C^47^, and C=C respectively. The weak and sharp peak at 1399 cm^−1^ can be ascribed to C=C^41^, stretching vibration. The strong and sharp peaks at 1244 cm^−1^, 1159 cm^−1^, and 828 cm^−1^ can be associated with C–O^48^, C–O^49^ and C–C^50^ stretching vibrations respectively. The peaks at 1305 cm^−1^ and 1030 cm^−1^ can be affiliated with the –C–O–C– stretching vibrations^48,51^. Similar peaks at 3287 cm^−1^, 1640 cm^−1^, 1599 cm^−1^, 1244 cm^−1^ and 828 cm^−1^ were observed for the plant extract which correspond to O–H, C=O/C=C, C=C, C–O and C–C respectively. Both the sample exhibit similar peaks, since the CQDs are developed from the same extract and possess common functional groups. This reveals, both CQDs and plant extract are rich in characteristic functional groups such as hydroxyl (-OH), carbonyl (> C=O), carboxyl (–COO^−^) and ether (–C–O–C–) groups, which aid in biocompatibility of the CQDs and enhance their potential in biological applications.

### In vitro* biomedical applications*

#### Cytotoxicity and cell viability test

**Figure 5:**
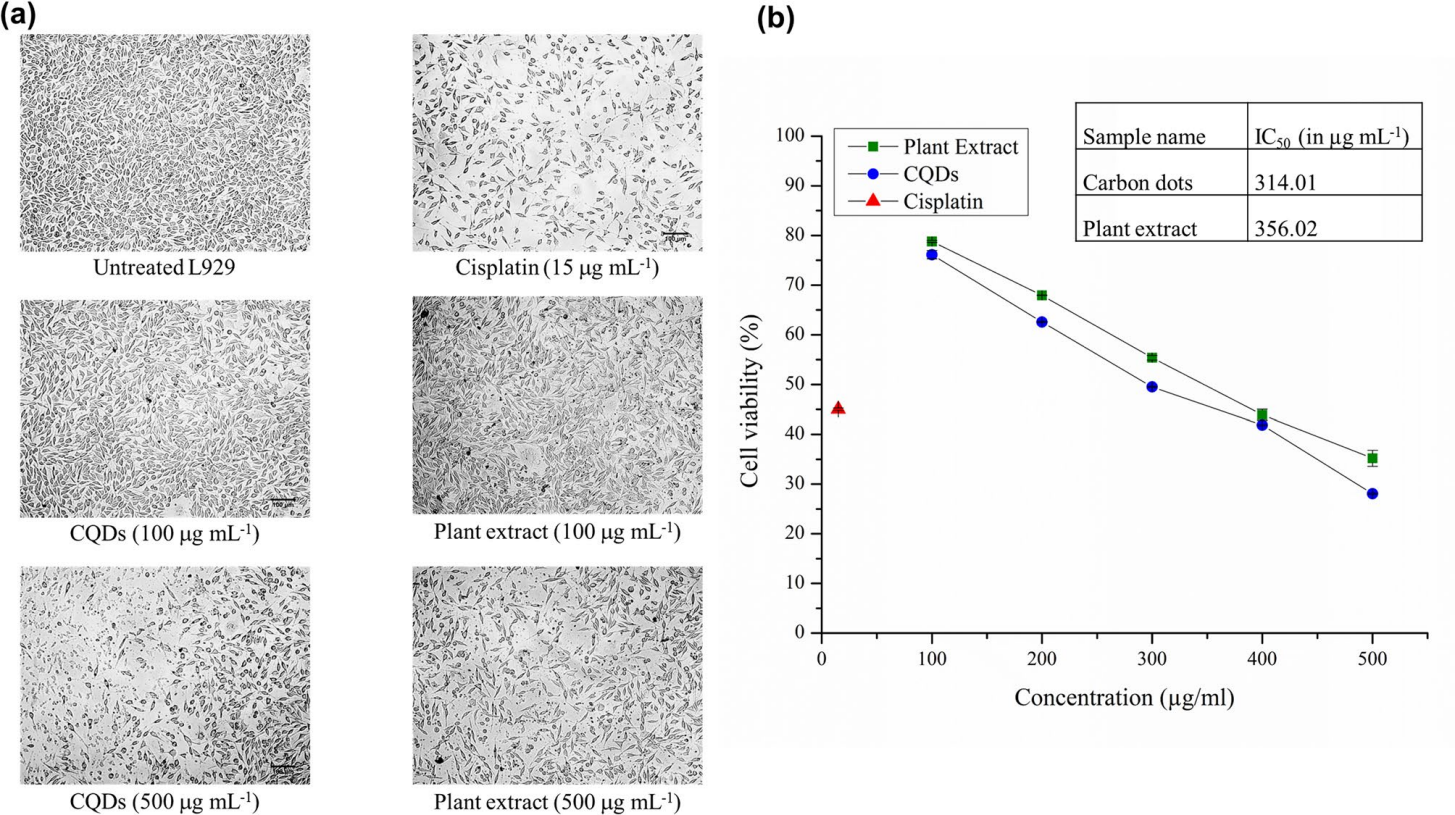
MTT assay using L929 fibroblast cell line: (**a**) images of cells and (**b**) graph representing the cytotoxic effect of different concentrations of CQDs, plant extract and control, cisplatin (15 µg mL^-1^) after treatment for 24 h. The results were expressed as mean ± SEM, n = 3.

Cytotoxicity and cell viability altering properties of CQDs and plant extract were evaluated by MTT assay using fibroblastic L929 (Fig. 5 and Fig. S1) and keratinocytic HaCaT (Fig. S2) cell lines. In the case L929 cells, cell viability effect of the plant extract was found to be ~ 78% at 100 μg mL^−1^ and ~ 35% at highest concentration (500 μg mL^−1^) with respect to untreated control. Comparatively, CQDs yielded viability of ~ 76% at 100 μg mL^−1^ and ~ 28% at 500 μg mL^−1^, while treatment of the standard drug cisplatin at 15 μg mL^−1^ resulted in ~ 45% cell viability (Table S1). The LC_50_ values of the CQDs and plant extract were calculated to be 314.01 and 356.02 μg mL^−1^ respectively, using the graph plotted (Fig. 5b). Interestingly, in HaCaT cells, CQDs were found to produce a cell viability of ~ 60% at the highest studied concentration of 1 mg mL^−1^, with a projected LC_50_ of 1660 µg mL^−1^. From these findings, it can be concluded that both plant extract and CQDs exhibit dose-dependent cytotoxicity effects on L929 fibroblast cells with the CQDs comparatively toxic than the plant extract, while the CQDs were non-toxic to HaCaT cells. Similarly, CQDs developed by from *Catharanthus roseus* leaves, at a concentration of 1 mg mL^−1^ exhibited 88 and 82% of cell viability rate in MCF-7 and MCF-10a cell lines respectively^52^.

**Figure 6:**
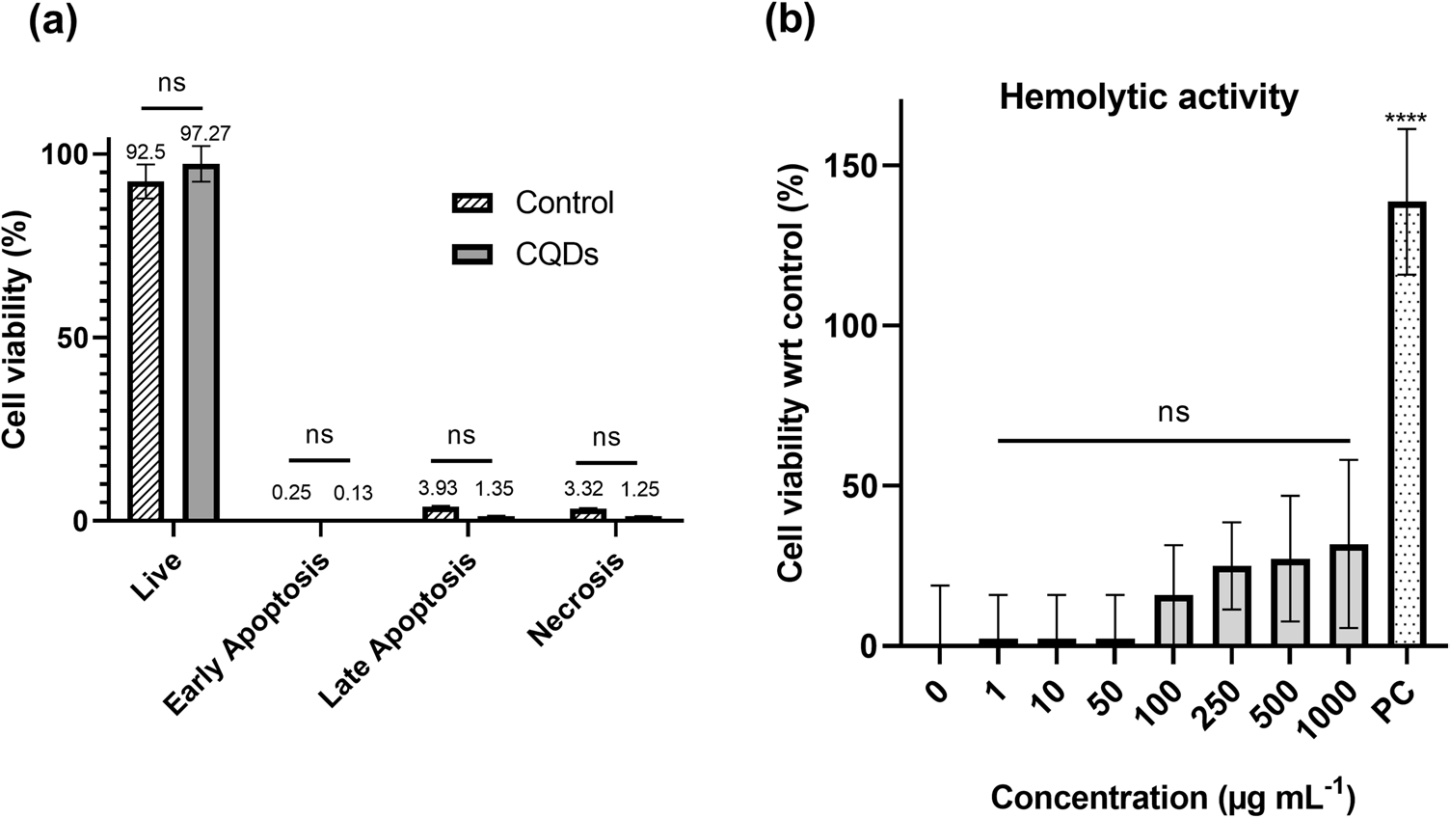
Apoptotic and hemolytic effect of CQDs: (**a**) Apoptotic and necrotic conditions of CQDs treated vs untreated (control) HaCaT cells; (**b**) Hemolytic activity of different concentrations of CQDs, PC = positive control (10% RBC lysis buffer). The results were expressed as mean ± SD, (^*ns*^*p* > 0.05, ^****^p < 0.0001, n = 4).

Further, the reason for cell death was studied via apoptosis assay using HaCaT via Annexin V and PI staining followed by flow cytometry. The Annexin V-FITC vs PI-PE graph (Fig. S3) led to the conclusion that the CQDs did not induce neither apoptosis nor necrosis, rather they protect the cells from cellular death as compared with control (untreated). Of the 1 × 10^6^ CQDs treated cells, 97.27% were living, while only 0.13, 1.35 and 1.25% cells entered early apoptotic, late apoptotic and necrotic phases respectively. However, 92.5, 0.25, 3.93 and 3.32% of the control cells were alive, in early apoptosis, late apoptosis and necrosis respectively (Fig. 6a).

The hemolytic activity of the CQDs was also evaluated using goat blood. CQDs exhibited minimal hemolytic activity in a dose dependent manner (Fig. 6b) with highest hemolysis of 31.82% at 1000 µg mL^−1^. The IC_50_ was calculated using log (inhibitor) vs normalized response variable slope fit to give of 3170 µg mL^−1^. This can imply that CQDs are not toxic towards RBCs. Most of CQDs synthesized are reported to possess least hemolytic effects, such as those prepared from BSA^53^ and p-phenylenediamine and polyethyleneimine^54^.

#### Wound healing property

**Figure 7:**
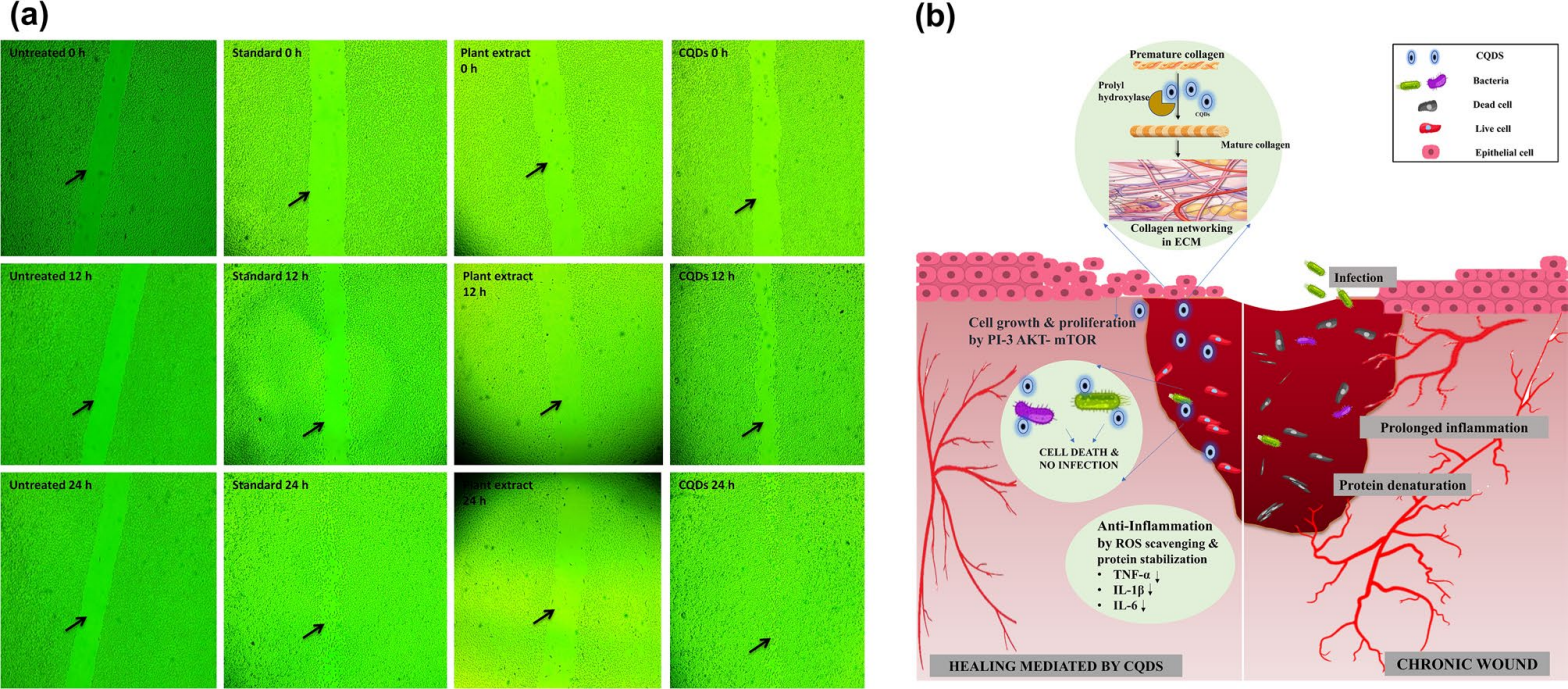
Wound healing property of CQDs: (**a**) The scratch healing assay of CQDs, plant extract and ascorbic acid (standard) at the concentration of 31.4, 35.6 and 15 µg mL^-1^ respectively, after 12 and 24 h, in L929 cells; (**b**) possible mechanism by which wounds are healed by CQDs.

Wound healing property of CQDs was examined through scratch assay (Fig. 7a and Fig S4). The dosages for CQDs and plant extract were determined based on the findings of the cytotoxicity assay. 10% of the LC_50_ value is a viable concentration that ensures safety in terms of toxicity. Hence, for L929 cells, 31.4 µg mL^-1^ and 35.6 µg mL^-1^ of CQDs and plant extract were chosen. The percentage of wound closure in 12 h for plant extract, CQDs and standard ascorbic acid were found to be 3.8, 51.05 and 41.81% respectively, which increased to 6.62, 99.87 and 99.68% respectively in 24 h, (Tables S2 and S3). In HaCaT, because of their non-toxic nature, a concentration of 250 µg mL^−1^ CQDs was used and was found to induce a wound closure of 57.59% while untreated cells recover 39.45%.

Collagen serves as the predominant constituent within the extracellular matrix (ECM), that plays a pivotal role in offering structural reinforcement to tissues and organs. Ascorbic acid, a cofactor for prolyl hydroxylase enzyme, assists in the hydroxylation of proline residues on alpha helices to form mature collagen^55^. Also, *H. rosa-sinensis* flower extract has been documented to increase the hydroxyproline content, indicating the formation of collagen, which can enhance tissue repair by promoting the formation of stable collagen fibrils^47^. Furthermore, CQDs can influence positively in the wound healing and cell growth signalling pathway such as PI3K/Akt/mTOR^56^, wherein, mTOR is reported to enhance the expression of collagen and other extracellular proteins^57^. The possible mechanism of wound healing of CQDs has been illustrated in Fig. 7b.

#### Anti-inflammatory activity

**Figure 8:**
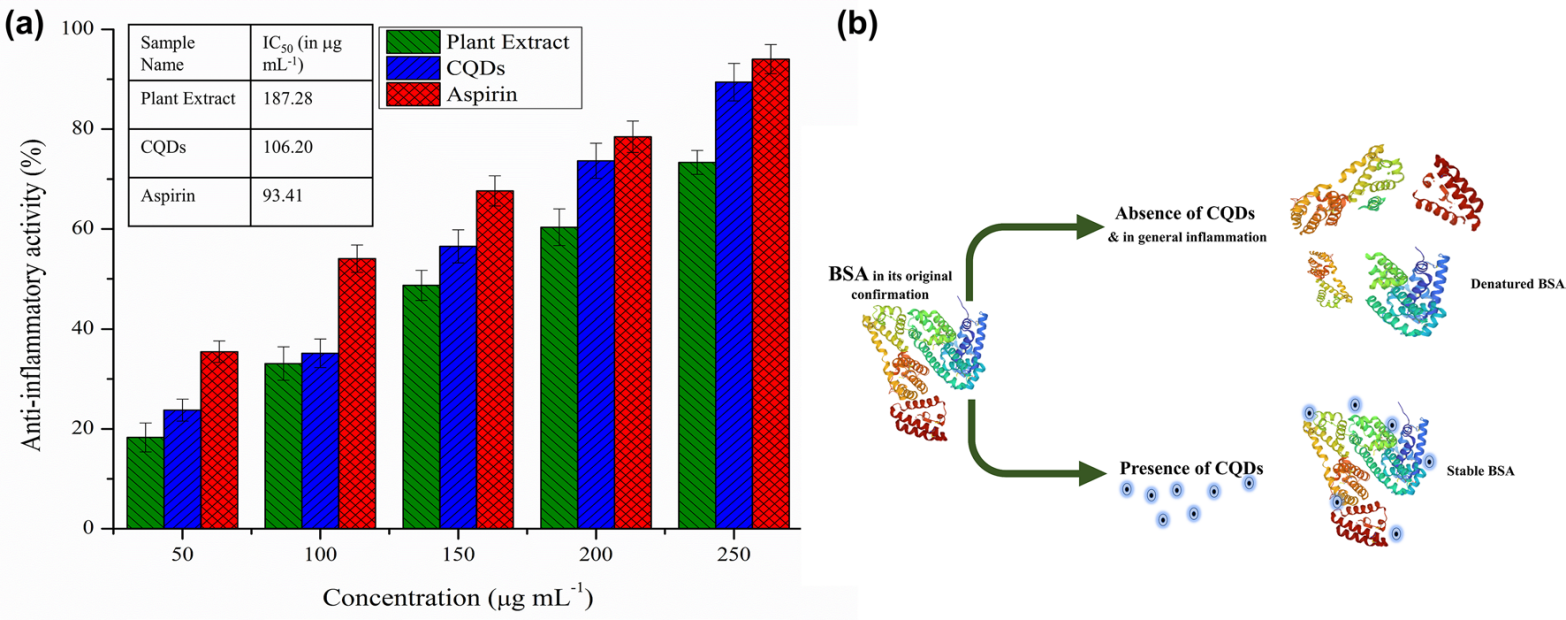
Protein denaturation inhibition (**a**) by varying concentrations of CQDs and plant extract compared with standard aspirin, along with their IC_50_ values (inset); (**b**) mechanism of inhibition of protein denaturation by CQDs. The results are expressed as mean ± SEM, n = 3.

The inflammatory action involves heat production due to vasodilation which also brings various chemical mediators for inflammation which reduces the local pH resulting in the protein denaturation. Protein denaturation assay was opted as an initial study to scrutinize the anti-inflammatory efficacy of *H. rosa-sinensis* extract and its CQDs against heat treated BSA (Fig. 8a). Both the samples exhibited profound anti-inflammatory response equivalent to that of the standard non-steroidal anti-inflammatory drug (NSAID), aspirin (IC_50_ = 93.41 μg mL^−1^). However, the CQDs were found to be more effective in inhibiting protein denaturation with an IC_50_ value of 106.20 μg mL^−1^ as compared to the plant extract (IC_50_ = 187.28 μg mL^−1^) (Table S4). Comparatively, Kasouni et al.^58^ described the anti-inflammatory activity of N-doped Carbon nanodots, which was able to inhibit the protein denaturation by 55% at its lowest (25 μg mL^−1^) and 97% at its highest concentrations (200 μg mL^−1^) respectively.

The inhibition of protein denaturation in vitro could be due to the binding of the CQDs to the native protein, via hydrogen bonds and van der Waal’s forces, which helps in stabilizing their structure from denaturation due to thermal stress, as depicted in Fig. 8b. However, the protein denaturation assay serves as an initial indication of the anti-inflammatory properties of the synthesized CQDs.

**Figure 9:**
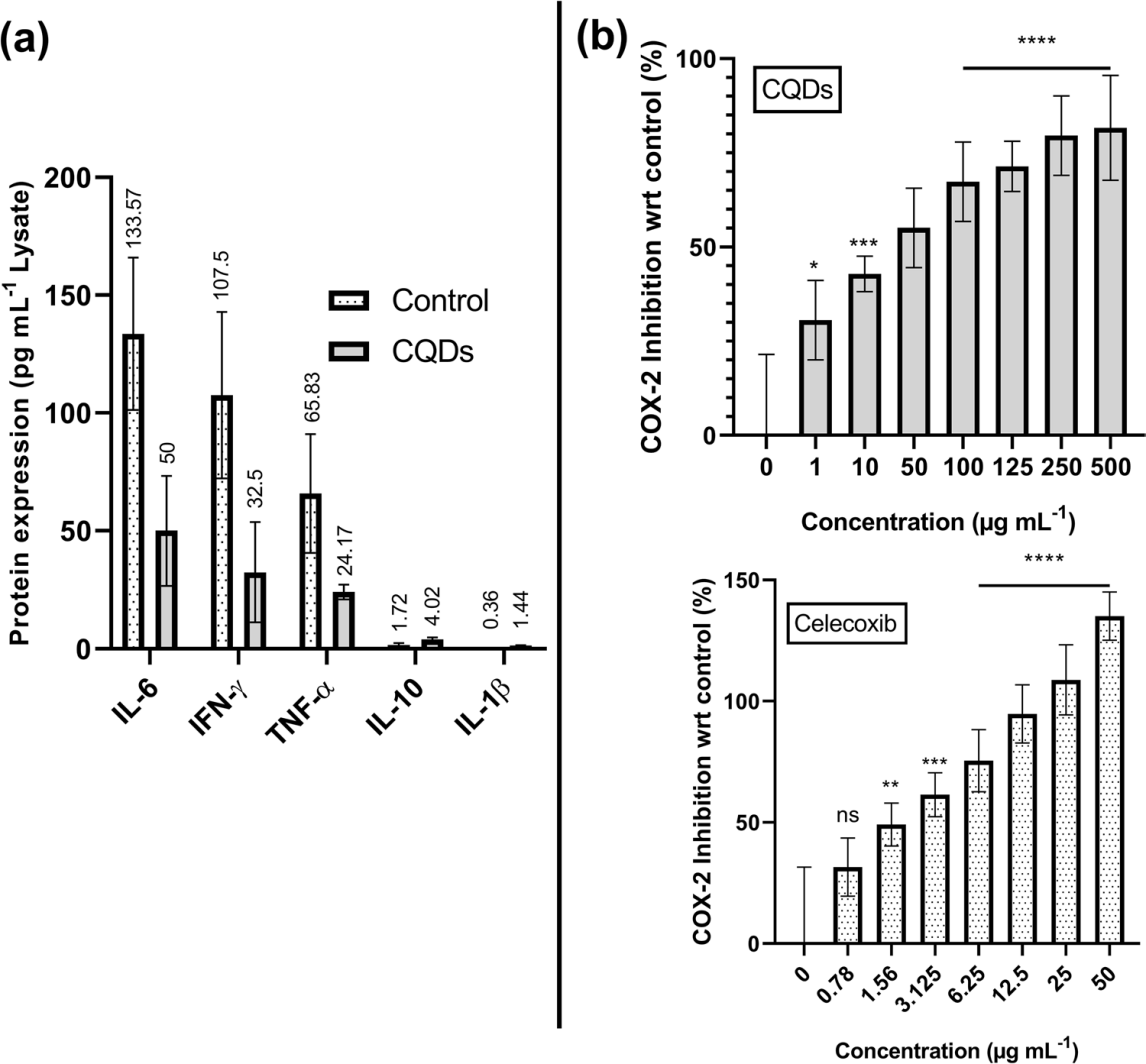
Anti-inflammatory activity of CQDs by (**a**) regulation of pro- and anti-inflammatory cytokines; (**b**) inhibition of COX-2, as compared with that of celecoxib. Results are expressed as mean ± SD (, (^*ns*^*p* > 0.05, *p < 0.05, **p < 0.01, ***p < 0.001, ****p < 0.0001, n = 4)).

Further corroboration of anti-inflammatory effects of CQDs were done via ELISA of different pro-inflammatory and anti-inflammatory cytokines using HaCaT cells. CQDs have been found to drastically decrease the production of pro-inflammatory cytokines TNF-α, IL-6 and INF-γ from 65.83, 133.57 and 107.50 pg mL^−1^ (in untreated control cells) to 24.17, 50.00 and 32.50 pg mL^−1^ respectively, at the same time slightly increasing the expression of pro- and anti-inflammatory cytokines IL-1β and IL-10 from 0.36 and 1.72 pg mL^−1^ to 1.44 and 4.02 pg mL^−1^ respectively (Fig. 9a), hence ensuring its anti-inflammatory capabilities. Similar findings have been reported in which the anti-inflammatory action of CQDs can be explained through their reactive oxygen species (ROS) scavenging activity which helps in minimizing the abundance of pro-inflammatory cytokines like IL-6, IL-1β and TNF-α^59^.

COX isozyme facilitates the transformation of arachidonic acid into prostaglandin H2 (PGH2), which is subsequently metabolized into pro-inflammatory prostaglandins such as prostaglandin E2 (PGE2), promoting vasodilation, increasing vascular permeability, and sensitizing sensory nerve endings, all contributing to inflammation^60^. The predominantly constitutive form, COX-1, is expressed throughout the body and provides homeostatic functions, while COX-2, the inducible form, gets expressed in response to inflammatory stimuli, such as cytokines, growth factors, or physical injury^61^. Most NSAIDs have an inhibitory action on the production of COX, with celecoxib being specific to COX-2^62^. As such, COX-2 inhibitory properties of the CQDs were checked and compared with that of celecoxib. It was found that CQDs inhibited COX-2 with an IC_50_ of 14.30 µg mL^−1^, while celecoxib had an IC_50_ of 1.79 µg mL^−1^ (Fig. 9b). From the study, we understand that CQDs inhibit COX-2 and regulate inflammatory cytokines to control inflammation.

Okulik et al.^63^ revealed the significant role of carboxylic acid of NSAIDs which forms an effective interaction with the enzyme thereby rendering its action leading to the reduction in prostaglandins. Similarly, the carboxylic acid moieties on the CQDs’ surface might interact with COX leading to the reduce in inflammation.

In a typical wound healing process, inflammation plays a critical role, where the outcome is directed by the balance between pro-inflammatory and anti-inflammatory factors. The homeostasis between pathways is explained by Bennett et al., that these antagonist pathways are influenced by cytokines and hormones. For example, IL-6, IL-1β, TNF-α which is inhibited by IL-10 and TGF-β which act contextually as pro- and anti-inflammatory cytokines respectively^64^.

#### Antimicrobial analysis

**Figure 10:**
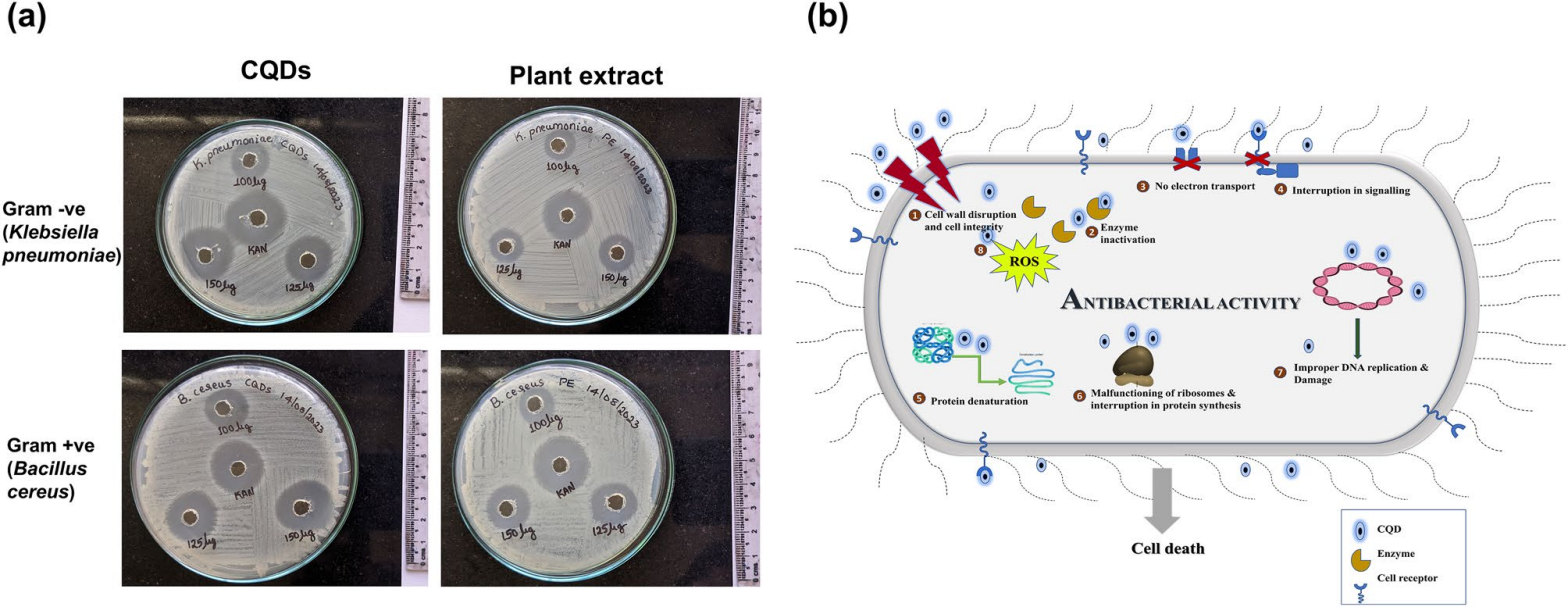
Antimicrobial activity of (**a**) different doses (100, 125 and 150 µg) of CQDs and plant extract against Gram-positive (*Bacillus cereus*) and Gram-negative (*Klebsiella pneumoniae*) bacteria using kanamycin (KAN—50 µg) as positive control; (**b**) possible mechanism of the antibacterial action of CQDs.

Antibacterial activity of CQDs were examined against Gram positive- *Bacillus cereus* and Gram negative- *Klebsiella pneumoniae* bacteria by means of well diffusion assay. Different concentration of *H. rosa-sinensis* plant extract and CQDs were allowed to diffuse in the agar wells and interact with the bacterial inoculums. Zones of inhibition were observed (Fig. 10a) in both bacteria at all doses of plant extract and CQDs respectively (Table S5). The plant extract developed inhibitory zones of 15–19 mm and 13–17 mm, while the CQDs showed zones of 19–24 mm and 16–23 mm, against *B. cereus* and *K. pneumoniae* respectively. Moreover, CQDs, at higher dose (150 μg), exhibited profound antimicrobial activity comparable with that of the standard kanamycin. Further, the colonies formed after 12 h incubation with the specific concentrations of CQDs, plant extract and kanamycin are shown in Fig. S5. When incubated with 150 mg mL^−1^ of CQDs, minimal formation of colonies was observed for both Gram-positive and Gram-negative bacteria. The highest concentration of CQDs used in this analysis can be considered safe as it is ~ 50% of the LC_50_ against L929 obtained from MTT assay. The antibacterial properties could possibly be due to the small size and less negative surface charge of the CQDs, which aids in binding to the bacterial surface, creating an electrostatic interaction, and leading to cell death via cell wall disruption, enzyme inactivation, interruption of electron transport and cell signalling, protein denaturation, ribosome disassembly, ROS generation, and/or DNA damage (Fig. 10b).

## Conclusion

This work combines the medicinal values of *Hibiscus rosa-sinensis* and unique attributes of CQDs, where CQDs were synthesized from the leaves of *H. rosa-sinensis* using microwave-assisted bottom-up approach. The synthesized quasi-spherical CQDs, with good fluorescent properties, possess compelling wound healing and anti-inflammatory capabilities as demonstrated through in vitro scratch healing and protein denaturation assays respectively. These claims were further supported by studies that revealed that the CQDs inhibit COX-2 and regulating pro- and anti-inflammatory cytokines, mainly responsible for the inflammation and wound healing. These CQDs also showcased very good biocompatibility with RBCs, skin and fibroblastic cells. Further, these CQDs exhibited strong antimicrobial activity against both *K. pneumoniae* and *B. cereus*. However, further detailed in vivo studies are necessary to understand its mechanism of action and efficacy properly. In conclusion, the bioinspired CQDs synthesized from *H. rosa-sinensis* leaves possessed good wound healing and antibacterial properties in vitro, paving the way for scope in the field of biomedicine when explored further.

### Supplementary Information

Supplementary Information.

## Data Availability

The data supporting the findings of this study are available within this article and its Supplementary Information Files. All other relevant data will be available from the corresponding author upon reasonable request.
