# Supplementary material for: Microwave-assisted synthesis, characterization and in vitro biomedical applications of Hibiscus rosa-sinensis Linn.-mediated carbon quantum dots
Source: Sci Rep. 2024 Apr 30;14:9915. doi: 10.1038/s41598-024-60726-y (PMC11061284; doi:10.1038/s41598-024-60726-y)
Supplement: Supplementary file 1 — Supplementary Information. [file 41598_2024_60726_MOESM1_ESM.docx]

**Microwave-Assisted Synthesis, Characterization and *in vitro* Biomedical Applications of *Hibiscus rosa-sinensis* Linn.-mediated Carbon Quantum Dots**

Shweta Yalshetti^1^, Bothe Thokchom^1^, Santosh Mallikarjun Bhavi^1^, Sapam Riches Singh^1^, Sneha R. Patil^1^, B. P. Harini^2^, Mika Sillanpää^3^, J. G. Manjunatha^4^, B. S. Srinath^5^, Ramesh Babu Yarajarla^1^*

^1^Drosophila and Nanoscience Research Laboratory, Department of Applied Genetics, Karnatak University, Dharwad, Karnataka 580003, India.

**^2^**Department of Zoology, Bangalore University, Bangalore, Karnataka 560056, India.

**^3^**Department of Biological and Chemical Engineering, Aarhus University, Norrebrogade 44, 8000 Aarhus C, Denmark.

**^4^**Department of Chemistry, FMKMC College, Mangalore University Constituent College, Madikeri, Karnataka 571201, India.

**^5^**Department of Microbiology and Biotechnology, Bangalore University, Bangalore, Karnataka 560056, India.

***Corresponding author:** Ramesh Babu Yarajarla

Drosophila and Nanoscience Research Laboratory, Department of Applied Genetics, Karnatak University, Dharwad, Karnataka 580003, India

E-mail: [rameshy@kud.ac.in](mailto:rameshy@kud.ac.in)

**
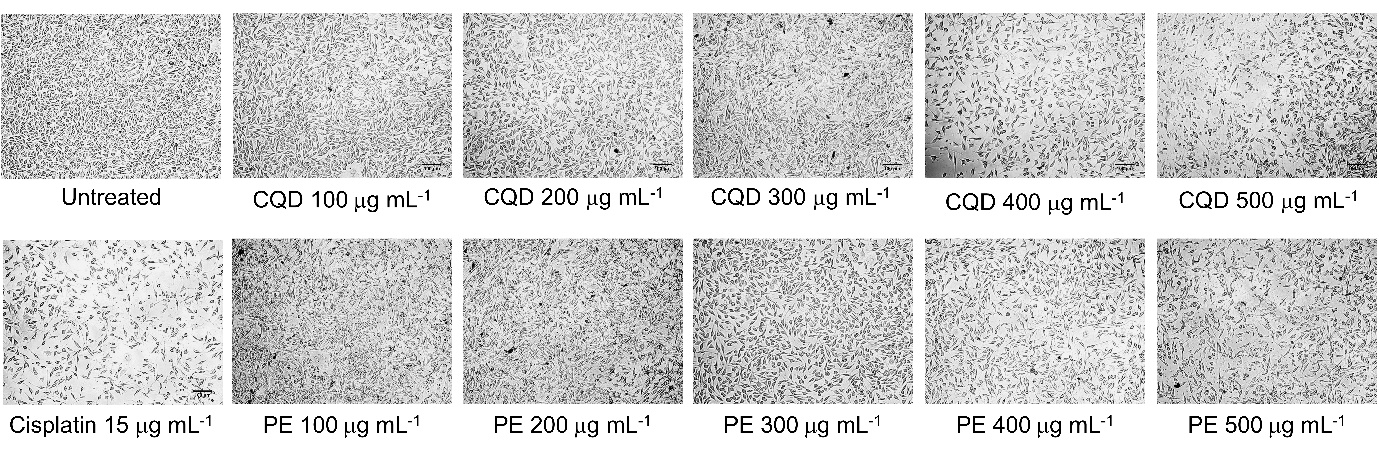
**

**Figure S1:** MTT assay images of L929 cells treated with different concentrations of CQDs and plant extract, with cisplatin as standard control; PE stands for plant extract


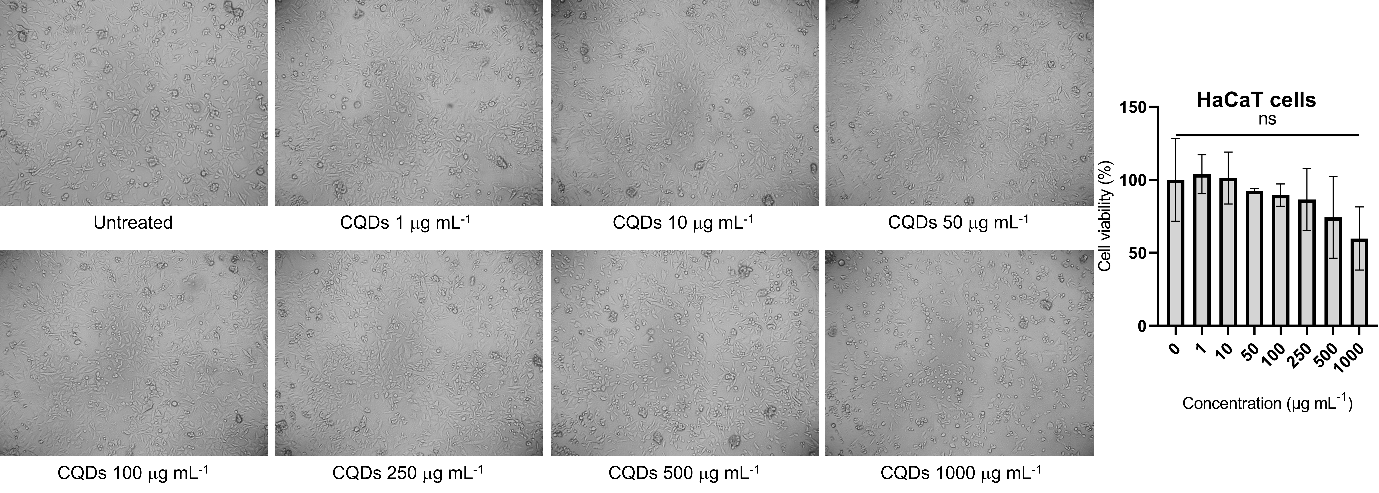


**Figure S2:** MTT assay images of HaCaT cells treated with different concentrations of CQDs along with bar graph. Bars represent mean ± SD (*^ns^p* > 0.05, n = 4)


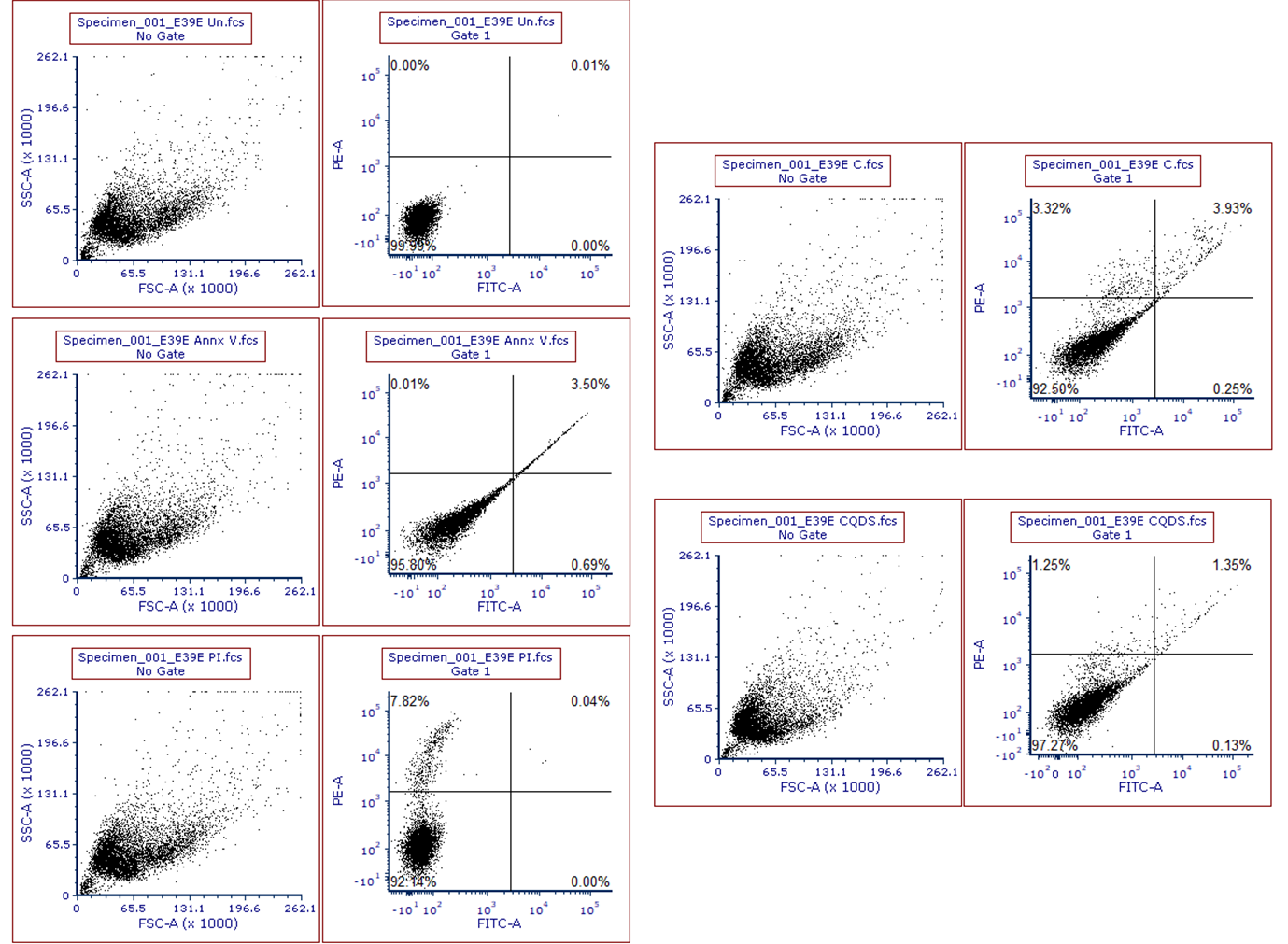


**Figure S3:** Flow cytometry dot plots (FSC vs SSC and Annexin V-FITC vs PI) of unstained, Annexin V-FITC only stained, PI only stained, control (untreated) and CQDs treated HaCaT cells


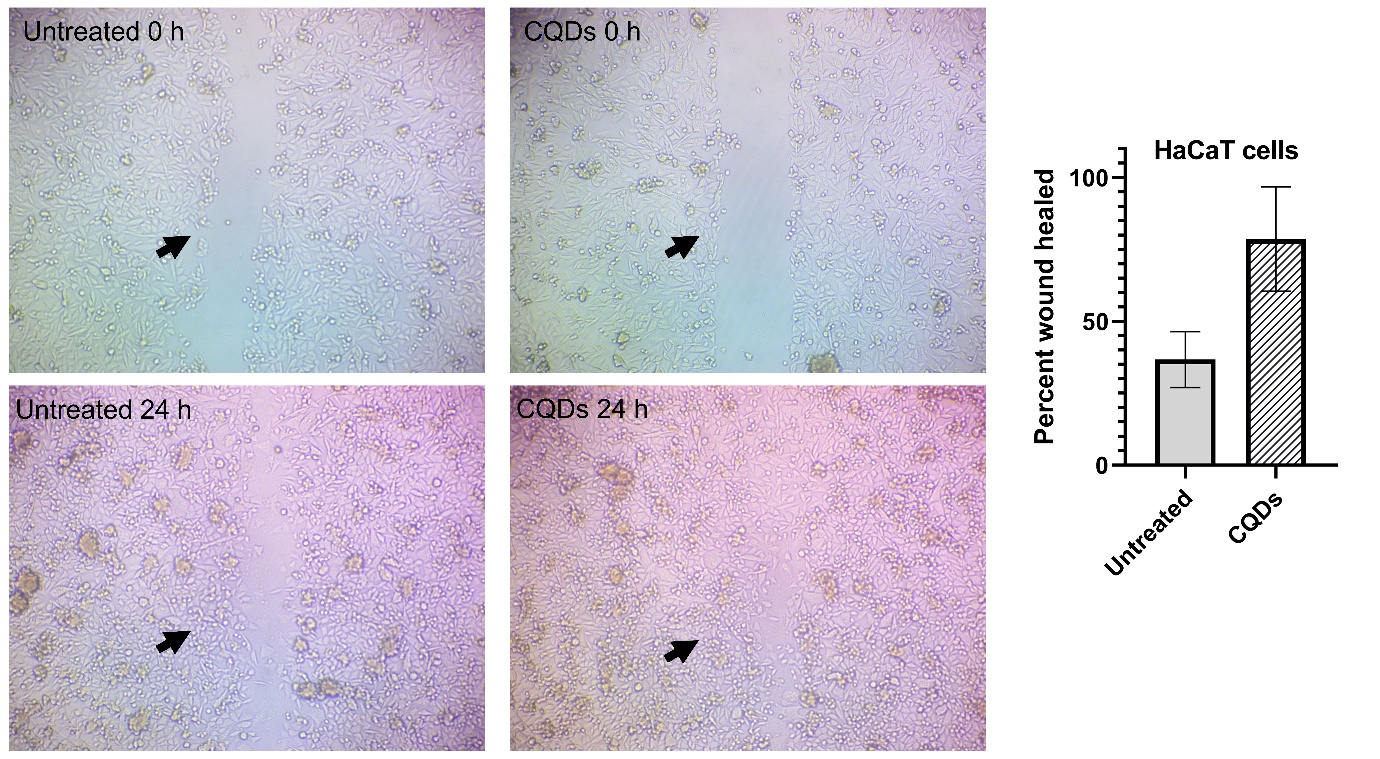


**Figure S4:** Scratch wound healing of untreated and 250 μg mL^-1^ CQDs treated HaCaT cells after 24 h and its respective graph. Bars represent mean ± SD, n = 3


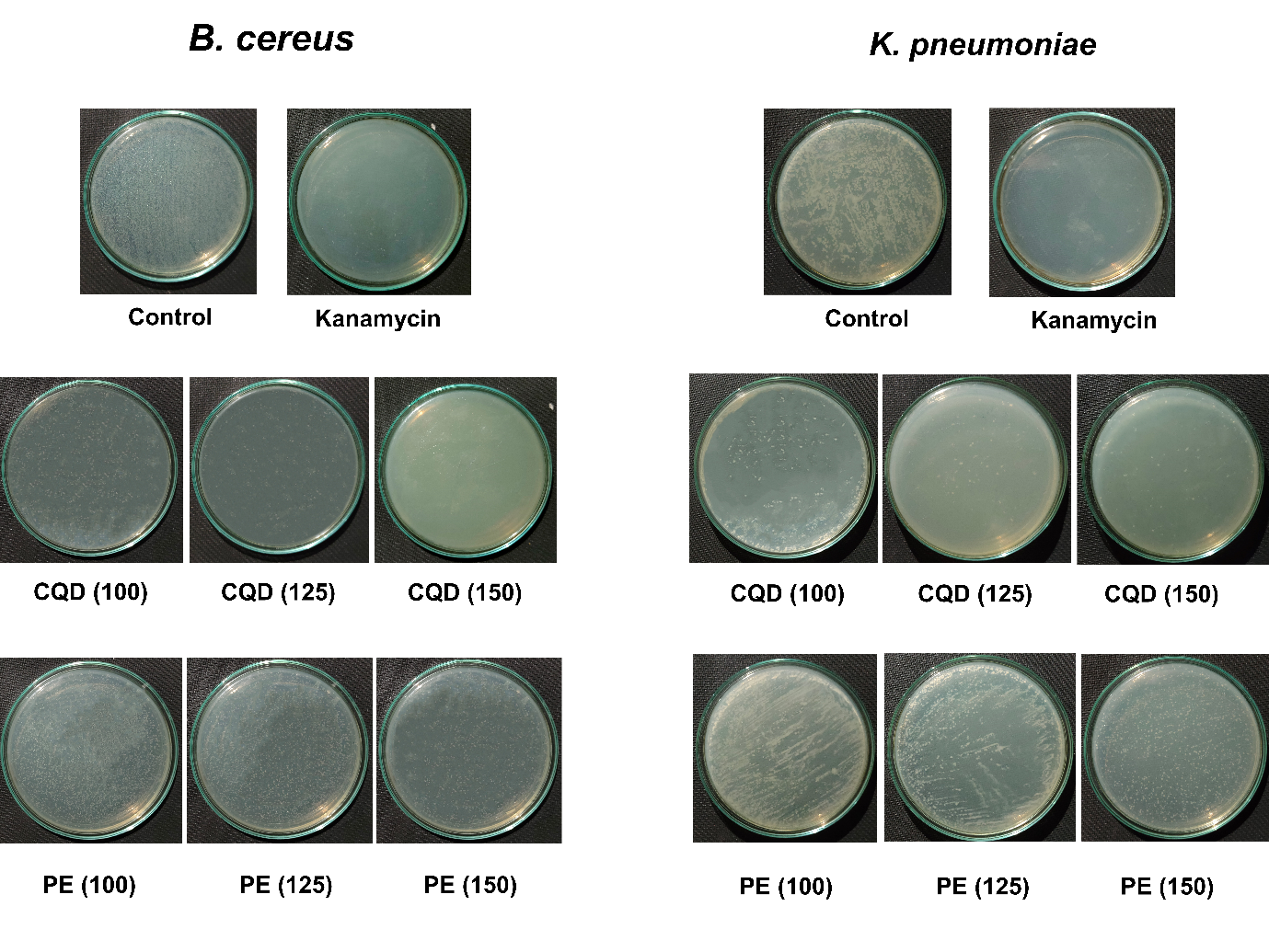


**Figure S5:** Colony counting assay: colonies of *B. cereus* and *K. pneumoniae* after 12 h culture with specific concentrations of CQDs, PE and 50 μg mL^-1^ kanamycin. PE = plant extract; (100), (125) and (150) represent the concentration of CQDs and plant extract in μg mL^-1^

**Table S1:** Cytotoxicity of CQDs against L929 cells compared with plant extract

| **Sample** | **Concentration (μg mL^-1^)** | **Cytotoxicity test by MTT assay** | |
| --- | --- | --- | --- |
|  |  | **Cell viability (%)** | **IC_50_ (μg mL^-1^)** |
| **CQDs** | 100 | 76.12 ± 0.1064 | 314.01 |
|  | 200 | 62.58 ± 0.6385 |  |
|  | 300 | 49.51 ± 0.0461 |  |
|  | 400 | 41.85 ± 0.0532 |  |
|  | 500 | 28.08 ± 0.0355 |  |
| **Plant extract** | 100 | 78.80 ± 0.1596 | 356.02 |
|  | 200 | 67.95 ± 0.0320 |  |
|  | 300 | 55.37 ± 0.2483 |  |
|  | 400 | 43.92 ± 0.4966 |  |
|  | 500 | 35.19 ± 0.5676 |  |
| **Cisplatin** | 15 | 45.02 ± 0.1064 | - |

^The results were analysed using one-way ANOVA (p < 0.05) and are expressed as mean ± SEM, n = 3^

**Table S2:** Data of wound closure analyzed using ImageJ

| **Cell** | **Label** | **Area (unit^2^)** | **Area %** | **Width (unit)** | **Standard deviation (unit)** | **Wound Closure %** |
| --- | --- | --- | --- | --- | --- | --- |
| L929 | Untreated 0 h | 0.625 | 10.006 | 0.246 | 0.019 | 0 |
|  | Untreated 12 h | 0.62 | 9.919 | 0.245 | 0.011 | 0.8 |
|  | Untreated 24 h | 0.618 | 9.896 | 0.242 | 0.011 | 1.12 |
|  | Standard 0 h | 0.928 | 14.847 | 0.371 | 0.02 | 0 |
|  | Standard 12 h | 0.54 | 8.645 | 0.221 | 0.045 | 41.81034 |
|  | Standard 24 h | 0.003 | 0.053 | 0.023 | 0.023 | 99.67672 |
|  | Plant Extract 0 h | 0.921 | 14.737 | 0.365 | 0.031 | 0 |
|  | Plant Extract 12 h | 0.886 | 14.173 | 0.357 | 0.04 | 3.800217 |
|  | Plant Extract 24 h | 0.86 | 13.76 | 0.346 | 0.033 | 6.623236 |
|  | CQDs 0 h | 0.764 | 12.223 | 0.306 | 0.028 | 0 |
|  | CQDs 12 h | 0.374 | 5.983 | 0.156 | 0.048 | 51.04712 |
|  | CQDs 24 h | 0.001 | 0.016 | 0.015 | 0.012 | 99.87 |
| HaCaT | Untreated 0 h | 1075006 | 10.915 | 411.555 | 122.306 | 0 |
|  | Untreated 24 h | 680623 | 6.911 | 266.628 | 105.242 | 36.6866 |
|  | CQDs 0 h | 1349017 | 13.697 | 490.372 | 56.709 | 0 |
|  | CQDs 24 h | 289011 | 2.934 | 189.878 | 87.412 | 78.5761 |

**Table S3:** Percentage wound closure observed in L929 Cell line

| **Cell line** | **Treatment** | **Concentration**  **(In µg mL^-1^)** | **Wound Closure at 12 h**  **(%)** | **Wound Closure at 24 h**  **(%)** |
| --- | --- | --- | --- | --- |
| L929 | Untreated | 0 | 0.8 | 1.12 |
|  | Standard Ascorbic Acid | 15 | 41.81 | 99.68 |
|  | Plant Extract | 35.60 | 3.8 | 6.62 |
|  | Carbon Quantum Dots | 31.40 | 51.05 | 99.87 |
| HaCaT | Untreated | 0 | - | 39.45 |
|  | Carbon Quantum Dots | 250 | - | 57.59 |

**Table S4**: Anti-inflammatory effect of CQDs compared with plant extract and aspirin

| **Sample** | **Concentration (μg mL^-1^)** | **Anti-inflammatory assay by protein denaturation** | |
| --- | --- | --- | --- |
|  |  | **% of inhibition** | **IC_50_ (μg mL^-1^)** |
| **CQDs** | 50 | 23.78 ± 2.2001 | 106.20 |
|  | 100 | 35.15 ± 2.8596 |  |
|  | 150 | 56.51 ± 3.3064 |  |
|  | 200 | 73.65 ± 3.5299 |  |
|  | 250 | 89.42 ± 3.7487 |  |
| **Plant extract** | 50 | 18.28 ± 2.9017 | 187.28 |
|  | 100 | 33.09 ± 3.3459 |  |
|  | 150 | 48.68 ± 3.0251 |  |
|  | 200 | 60.32 ± 3.6843 |  |
|  | 250 | 73.33 ± 2.3999 |  |
| **Aspirin** | 50 | 35.47 ± 2.1572 | 93.41 |
|  | 100 | 54.04 ± 2.7353 |  |
|  | 150 | 67.61 ± 3.0314 |  |
|  | 200 | 78.47 ± 3.1401 |  |
|  | 250 | 94.03 ± 2.908 |  |

^The results were analysed using one-way ANOVA (p < 0.05) and are expressed as mean ± SEM, n = 3^

**Table S5**: Antibacterial activity of CQDs and plant extract

| **Bacteria** | **Zone of Inhibition (in mm)** | | | | | | |
| --- | --- | --- | --- | --- | --- | --- | --- |
|  | **Kanamycin**  **(50 μg)** | **CQDs** | | | **Plant extract** | | |
|  |  | **100 μg** | **125 μg** | **150 μg** | **100 μg** | **125 μg** | **150 μg** |
| *Bacillus cereus* | 24.08 ± 0.3700 | 19.20 ± 0.4580 | 22.56 ± 0.6537 | 24.91 ± 1.2581 | 15.21 ± 0.2890 | 18.62 ± 0.8875 | 18.75 ± 0.1230 |
| *Klebsiella pneumoniae* | 23.82 ± 0.4966 | 16.77 ± 0.0261 | 19.96 ± 0.0367 | 22.57 ± 0.7426 | 13.86 ± 0.5754 | 15.45 ± 0.3685 | 16.99 ± 0.1363 |

^The results are expressed as mean ± SEM, n = 3^
